# Validity of Virtual Reality Body Exposure to Elicit Fear of Gaining Weight, Body Anxiety and Body-Related Attentional Bias in Patients with Anorexia Nervosa

**DOI:** 10.3390/jcm9103210

**Published:** 2020-10-05

**Authors:** Bruno Porras-Garcia, Marta Ferrer-Garcia, Eduardo Serrano-Troncoso, Marta Carulla-Roig, Pau Soto-Usera, Helena Miquel-Nabau, Nazilla Shojaeian, Isabel de la Montaña Santos-Carrasco, Bianca Borszewski, Marina Díaz-Marsá, Isabel Sánchez-Díaz, Fernando Fernández-Aranda, José Gutiérrez-Maldonado

**Affiliations:** 1 Department of Clinical Psychology and Psychobiology, University of Barcelona, Passeig de la Vall d’Hebron 171, 08035 Barcelona, Spain; brporras@ub.edu (B.P.-G.); martaferrerg@ub.edu (M.F.-G.); helena.mn29@gmail.com (H.M.-N.); nazila.shojaeian@gmail.com (N.S.); 2 Department of Child and Adolescent Psychiatry and Psychology, Hospital Sant Joan de Déu of Barcelona; Passeig de Sant Joan de Déu, 2, 08950 Esplugues de Llobregat, Spain; eserrano@sjdhospitalbarcelona.org (E.S.-T.); mcarulla@sjdhospitalbarcelona.org (M.C.-R.); psoto@sjdhospitalbarcelona.org (P.S.-U.); 3 Department of Psychiatry and Mental Health, Hospital Clínico San Carlos, Madrid, Calle del Prof Martín Lagos, s/n, 28040 Madrid, Spain; isabelmsc.is@gmail.com (I.d.l.M.S.-C.); biancbor@ucm.es (B.B.); marinadiaz.marsa@salud.madrid.org (M.D.-M.); 4 Department of Psychiatry and Mental Health, Hospital Universitario de Bellvitge- IDIBELL and CIBEROBN, Barcelona; Carrer Feixa Llarga s/n, 08907 Hospitalet del Llobregat, Spain; isasanchez@bellvitgehospital.cat (I.S.-D.); ffernandez@bellvitgehospital.cat (F.F.-A.)

**Keywords:** anorexia nervosa, virtual reality, fear of gaining weight, body anxiety, body image disturbances, body-related attentional bias

## Abstract

Fear of gaining weight (FGW), body image disturbances, associated anxiety and body-related attentional bias are the core symptoms of anorexia nervosa (AN) and play critical roles in its development and maintenance. The aim of the current study is to evaluate the usefulness of virtual reality-based body exposure software for the assessment of important body-related cognitive and emotional responses in AN. Thirty female patients with AN, one of them subclinical, and 43 healthy college women, 25 with low body dissatisfaction (BD) and 18 with high BD, owned a virtual body that had their silhouette and body mass index. Full-body illusion (FBI) over the virtual body was induced using both visuo-motor and visuo-tactile stimulation. Once the FBI was induced, the FBI itself, FGW, body anxiety and body-related attentional bias toward weight-related and non-weight-related body areas were assessed. One-way analyses of covariance (ANCOVA), controlling for age, showed that AN patients reported higher FGW, body anxiety and body-related attentional bias than healthy controls. Unexpectedly, patients with AN reported significantly lower FBI levels than healthy participants. Finally, Pearson correlations showed significant relationships between visual analog scales and body-related attentional bias measures, compared to other eating disorder measures. These results provide evidence about the usefulness of virtual reality-based body exposure to elicit FGW and other body-related disturbances in AN patients. Thus, it may be a suitable intervention for reducing these emotional responses and for easing weight recovery.

## 1. Introduction

Anorexia nervosa (AN) is considered one of the most serious eating disorders (ED), affecting women worldwide [1]. Although the age of onset of AN is established at around 14 to 19 years old [2,3], this condition has been progressively diagnosed in younger patients [4,5], making early preventive interventions in AN a public health priority. 

According to the Diagnostic and Statistical Manual of Mental Disorders, 5th Edition [6], individuals with AN show severe restriction of food intake that leads to a significantly low body weight. In addition, they usually feel an intense fear of gaining weight (FGW) and disturbances in the way they experience their body weight and shape, also referred to as body image disturbances [7]. Greater FGW has also been related to more severe ED symptoms [8,9], and it is considered as one of the strongest predictors of ED symptomatology (e.g., dietary restraint and compulsive exercise) in AN patients [10]. In fact, it has been suggested that AN patients are more susceptible to learning fear associations than healthy individuals [11] (Strober, 2004); for instance, they are more likely to develop an intense FGW after starting a low-fat diet. Another psychopathological model has highlighted the importance of the anxiety symptomatology that AN patients usually experience as a direct reinforcement of their fears and behaviors [12]. The subjective feelings of tension, worried thoughts and physical changes that AN patients present are sometimes focused on their own body or toward specific body areas (i.e., weight-related body areas), a phenomenon known as body anxiety [13]. Previous studies have suggested that the core fears in AN (such as FGW) elicit high anxiety levels, which, in turn, lead to a progressive increase in ritualistic eating- and activity-related behaviors (e.g., the initiation of noncaloric diets or doing intense physical exercise). Over time, this allows the reinforcement of undereating and other dysfunctional behavioral disturbances [12]. 

Other theories have focused on the study of the mechanisms underlying the information processing biases typically found among individuals with EDs: biases in memory, interpretation and attention of body-related information, which, in turn, influence and maintain body image disturbances and ED symptomatology [14,15]. Among these cognitive biases, the dysfunctional role of attention has received increasing consideration over the last decades. Previous research suggests that ED patients pay more attention to disorder-relevant information (e.g., food- and body-related stimuli) than to other sorts of information [16,17]. Furthermore, dysfunctional body-related attention presumably maintains body image disturbances by processing only body information that is consistent with dysfunctional cognitive schema content (such as my thighs or stomach are getting fatter), while schema-inconsistent information tends to be visually neglected [16]. When considering patients with AN, body-related attentional bias seems to be especially pronounced in restrictive-type AN patients [18].

Therefore, these body-related constructs and underlying cognitive mechanisms may play a critical differential role in the development and maintenance of AN symptomatology. Consequently, the implementation of specific, tailored interventions targeting them is needed in AN. 

Mirror exposure therapy aims to decrease body- and weight-related fears and is used to improve cognitive behavioral therapy (CBT) [19]. Several studies support the use of mirror exposure therapy to reduce body image disturbances in ED patients [20,21] and high body-dissatisfied individuals [22,23]. However, mirror exposure therapy frequently elicits a highly negative initial reaction in patients [24], which increases the probability of treatment rejection and the risk of dropout [25]. Furthermore, mirror exposure therapy is usually conducted in controlled settings (e.g., a laboratory or therapist’s office), and the generalization of positive changes learned during exposure is impaired [26].

These limitations can be overcome by the application of virtual reality (VR) techniques. For instance, VR technology is proposed as a good complement to in vivo exposure, especially in the initial stages of the intervention, since it is perceived as safer by participants and reduces the rates of withdrawal [27]. In addition, although VR is also conducted in a controlled setting, it allows researchers to create real-size 3D simulations of participants’ bodies with their physical characteristics and place them in immersive environments that reproduce real life situations related to their body image concerns (e.g., a dressing room, a beach or a bathroom), thus enhancing the generalization of positive results [26,28]. Another advantage of using VR is that it allows certain stimuli or environments to be manipulated in ways that may not be possible in mirror exposure therapy. For instance, it allows patients with AN to increase/decrease the weight (or the body mass index (BMI)) of the virtual avatar until it reaches a target weight, thus helping to confront their core fears (i.e., the fear of gaining weight). 

VR technology has evolved notably over the last few years. For instance, the newest VR systems can achieve a full-body motion tracking that captures the individual’s body silhouette and movements using a head-mounted display, VR controllers and a series of body trackers that can be attached to different body areas (e.g., the feet and the waist). These technological advances have allowed the development of countless applications for improving health-related problems in a new transdisciplinary research field known as embodied medicine or embodied technology (for an extensive review, see [29,30]). This paradigm allows individuals to perceive and feel a virtual body as their real body by activating predicting neurological brain circuits to elicit the feelings of ownership over a virtual avatar [29,30]. Specifically, the subjective experience in which individuals feel an artificial body to be their own is known as full-body illusion (FBI) [31]. To accomplish this, different types of information (visual, acoustic, proprioceptive and vestibular) must be combined into multisensory representations [32].

One of the most widely studied and best-known paradigms for eliciting feelings of ownership over a specific body area is the rubber hand illusion [33], in which the participant’s nonvisible real hand is synchronously touched by the experimenter while the participants see the same touches delivered to a fake rubber hand. Following the rubber hand illusion paradigm, a previous study found that ED patients tend to experience a stronger illusion over a fake hand than healthy individuals [34]. Alternatively, a large number of studies have investigated the effects of the FBI on the whole body to assess body image disturbances, for a review, see [28], finding that the perception of body size and weight may be changed in nonclinical samples [35,36,37,38] and ED patients [39,40,41]. Specifically, among AN patients, Keizer et al. [39] found that owning a skinny virtual body significantly reduced the perceived body size in AN patients, as well as in healthy participants. These results were later replicated by two case report studies, which showed that VR embodiment-based techniques (e.g., using a visuo-tactile stimulation procedure) allowed the modification of body image disturbances either in the short term [40] or over several sessions [41].

Curiously, while most body exposure-based therapies, such as mirror exposure therapy, focus on treating body image disturbances, little information is available about the use of body exposure techniques, with or without VR, to treat FGW. Levinson, Rapp and Riley [42] proposed an imaginal exposure procedure that consisted of five sessions in which the patient had to visualize her own weight gain and the possible associated adverse consequences. The authors reported a clinically significant change in ED symptomatology from pretherapy to one-month follow-up and a steady increase in weight throughout the exposure sessions, which was maintained at follow-up. Imaginal exposure therapy directly targeting the FGW seems to be an interesting approach in AN treatments. However, its effectiveness must be explored in controlled clinical studies. Furthermore, imagery exposure may have some significant limitations—for example, difficulty in achieving or maintaining visualization and the risk of avoidance of the most feared stimulus during visualization. These limitations can be overcome by the application of VR techniques that do not rely on the patient’s visualization ability and reduce the possibility of avoidance behaviors. Therefore, VR exposure therapy provides a useful approach to targeting FGW [41].

The aim of the current study was to assess the usefulness of a VR-based body exposure software to elicit FGW and other important body-related cognitive and emotional responses, such as body anxiety or body-related attentional bias among patients with AN. Specifically, the objective was to assess levels of FBI, body anxiety, FGW and body-related attentional bias when healthy women with high and low body dissatisfaction (BD) and AN patients owned a virtual body (VB) with their real silhouette and body mass index (BMI). In addition, the relationship between FGW, body anxiety and body-related attentional bias experienced during VR exposure and ED measures, collected prior to entering the VR, were assessed. Based on previous research, it was expected that: (i) AN patients would report higher levels of FBI, body anxiety and FGW than healthy women when they owned their real-size VB; (ii) AN patients would show higher body-related attentional bias, specifically in weight-related body areas, than healthy participants; (iii) healthy women with high BD would show higher FBI, body anxiety, FGW and body-related attentional bias than women with low BD; (iv) and there would be positive relationships between the measures assessed during VR exposure and the ED measures. 

## 2. Materials and Methods

### 2.1. Participants 

Forty-three female college students from the University of Barcelona, Spain and 30 AN female patients from different ED units in Barcelona participated in the study. College women were recruited through campus flyers and advertisements in social network groups. The exclusion criteria were a self-reported diagnosis of a current ED, a body mass index (BMI) of less than 17 (moderate thinness) or over 30 (obesity, according to the World Health Organization, 2004) or a self-reported current severe mental disorder diagnosis (e.g., schizophrenia or bipolar disorder).

The inclusion criteria were patients with a primary diagnosis of AN (DSM-5 criteria) over 13 years of age with a BMI < 19. Furthermore, one subsyndromal patient who met all anorexia criteria but two was also included. Visual deficits that prevent exposure, epilepsy, pregnancy and clinical cardiac arrhythmia were considered exclusion criteria for the nonclinical and clinical samples. 

Nineteen adolescents with AN were diagnosed at the ED Unit of Hospital Sant Joan de Déu of Barcelona, while 11 adults with AN were diagnosed at the ED Unit of Bellvitge Hospital and Hospital Clínico San Carlos of Madrid, Spain. All patients underwent a day-patient treatment for adolescents and young adults with ED. This was an intensive day-patient treatment program conducted at the ED Unit in 11-h periods, with permission to sleep at home.

### 2.2. Measures

#### 2.2.1. ED Symptomatology Measures

- BMI = weight (in kilograms)/ height (in m)².
- Eating Disorder Inventory (EDI-3) [43]. The EDI-3 is a self-reported inventory consisting of 12 scales and 91 items, in which the answers are provided on a 6-point Likert scale. In the current study, only the 10-item body dissatisfaction scale (EDI-BD) and 7-item drive for thinness (EDI-DT) scales were used. EDI-BD measures body dissatisfaction with the whole body and specific body parts. EDI-DT measures the desire to be thinner, concern with dieting, preoccupation with weight and FGW. The Spanish version of the EDI-3 has robust validity indices and good internal consistency, with a Cronbach’s alpha ranging from 0.74 to 0.96 [44]. In the current study, Cronbach’s alphas for the clinical samples were 0.76 for the EDI-BD scale and 0.84 for EDI-DT. Cronbach’s alpha values for both scales were 0.77 to 0.79 in the nonclinical sample.
- Physical Appearance State and Trait Anxiety Scale (PASTAS) [13]. The PASTAS is a self-reported questionnaire that assesses body anxiety, understood as the subjective feelings of tension, worried thoughts and physical changes that patients and participants feel about their body or toward specific parts of their body. The PASTAS comprises two self-report scales measuring weight-related and non-weight-related anxiety. In this study, the weight scale (W) with 8 items was used. The questionnaire presents good reliability, with a Cronbach’s alpha between 0.82 and 0.92 and good test-retest (*r* = 0.87) and convergent validity indices for the W scale (*r* = 0.74 EDI-BD and *r* = 0.62 EDI-DT) [13].
- Body Image Assessment Scale-Body Dimensions (BIAS-BD) [45]. The BIAS-BD was used to assess body image disturbances in this study. The BIAS is a figural drawing scale questionnaire, which presents the physical anthropometric dimensions of adult women in a series of human silhouettes. Participants selected the one that was perceived as their body size (perceived silhouette) and the one they desired to have (desired body size). Then, according to their BMI, the real silhouette was also selected. The scale allows researchers to estimate the participant’s body dissatisfaction (discrepancy between perceived and ideal body size) and body distortion (discrepancy between perceived and real body size). This scale shows good psychometric properties, such as good test-retest reliability (r = 0.86) with data collection before and after a two-week interval and good concurrent validity (r = 0.76) after comparing participants’ perceived actual body size and self-reported BMI [45].
- Body Appreciation Scale (BAS) [46], translated by Jáuregui-Lobera and Bolaños-Ríos [47]. The BAS consists of 12 items assessing a positive attitude towards one’s body on a 5-point Likert scale. It presents good internal consistency, with a Cronbach’s alpha of 0.908 [47].

#### 2.2.2. Body-Related Attentional Bias

**Figure 1:**
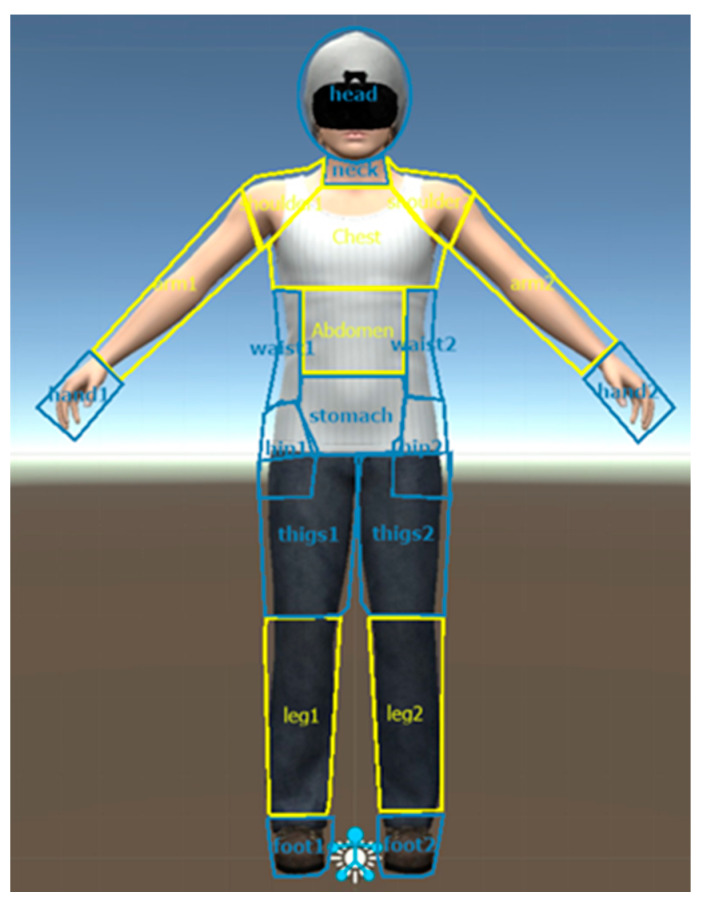
Weight-related and non-weight-related areas of interest on the female virtual avatar.

Weight-related body parts (W-AOIs or areas of interest) were chosen based on the weight scale of body items from the PASTAS questionnaire [13] and drawn onto a picture of a female avatar in a frontal view. Body parts included in the W-AOIs were the legs, thighs, buttocks, hips, stomach (abdomen) and waist. After the separation of the W-AOIs, the remaining body parts (head, neck, chest, shoulders, arms and feet) were labeled as non-weight-related body parts (NW-AOIs) (Figure 1). 

Participants’ selective visual attention was measured using the complete fixation time and number of fixations on the areas of interest (AOIs). Visual fixation was defined by Jacob and Karn [48] as the visual act of maintaining one’s gaze on a single location for a minimum duration, usually 100–200 ms. In this study, a duration of 100 ms was considered. Previous studies focusing on body-related attention used these specific gaze-behavioral measures, provided by eye-tracking (ET) technology, as reliable and continuous measures of attention allocation toward specific body areas [18,49,50,51,52].

#### 2.2.3. Visual Analog Scales (VAS)

- Full-body illusion (FBI) was assessed by means of a VAS estimating the intensity of the illusion from 0 to 100. “On a scale of 0 to 100, indicate to what extent you felt that the virtual body was your own body, where 0 is not at all and 100 is completely.”
- FGW and anxiety related to the whole body were assessed on a VAS from 0 to 100. “On a scale of 0 to 100, indicate the level of anxiety toward your body that you are feeling at this moment, where 0 is not at all and 100 is a lot.” “On a scale of 0 to 100, indicate to what extent you are afraid of gaining weight at this moment, where 0 is not at all and 100 is a lot.”

### 2.3. Hardware and Software Features

All participants were exposed to an immersive virtual scenario using a VR HTC-VIVE head-mounted display (HMD HTC VIVE, HTC Corporation, New Taipei City, Taiwan). In addition to the two controllers that HTC-VIVE usually provides, three additional body trackers were used to achieve full-body motion tracking. The VR trackers and headsets were connected to a computer with a powerful graphics card (Nvidia RTX 2080, Nvidia Corporation, Santa Clara, CA, USA) to run VR environments fluently.

HMD FOVE-Eye Tracking (FOVE, Inc., Torrance, CA, USA) was used to detect and register eye movements. The headset uses the incorporated position and orientation eye-tracking systems. The FOVE display has 2560*1440 pixels and creates 70 frames per second. Infrared eye-tracking sensors create 120 frames per second, with an accuracy level of less than 1 degree. FOVE setup 0.16.0 (FOVE, Inc., Torrance, CA, USA) and Unity 3D 3.0.0 (Unity Technologies, San Francisco, CA, USA) were used to create the virtual simulations. The virtual avatars were created using the software Blender 2.78. The virtual environment consisted of a room with a large mirror on the front wall. The mirror was large enough to reflect every limb of the body and was placed 1.5 m in front of the participants. A young female avatar wearing a basic white t-shirt with blue jeans and black trainers was created. The avatar also wore a swim cap to avoid any influence of hairstyle and an HMD, like the participants.

### 2.4. Procedure

This study was approved by the ethics committee of the University of Barcelona (Institutional review board IRB00003099), and all sessions were conducted at the ED units of each hospital. Furthermore, all AN patients were previously informed about the nature of the study by the clinicians responsible for their care at each ED unit. All participants signed the informed consent after being informed about the study, the data confidentiality and the possibility of withdrawing from the study at any point without consequences. When participants were under 18, informed consent was also signed by their legal guardians. Additionally, confidentiality was ensured by assigning a different identification code to each participant. Patients were previously diagnosed by senior trained clinical psychologists and psychiatrists using nonstructured clinical interviews and applying the DSM-5 criteria strictly. The Mini International Neuropsychiatric Interview (MINI) [53] was also conducted at two of the ED units. The assessment was carried out by trained clinical psychologists who had previous experience with the latest version of the MINI.

Before the start of the assessment session, each participant was measured to calculate their BMI, and the researchers asked questions in relation to the inclusion and exclusion criteria. In the ED units, the clinicians responsible for the patients were contacted to complete the identification form for the study, with information about current BMI, AN subtype diagnosis and previous history of the disorder, and to confirm that the inclusion and exclusion criteria were fulfilled. 

All the participants underwent the same procedure. Firstly, the virtual avatar was generated by taking frontal and lateral photos of the participant and creating an avatar whose silhouette matched the pictures by adjusting the body parts (shoulders, arms, chest, waist, stomach, hip, thighs and legs) to the photographs. In the meantime, a second researcher administered the paper-based questionnaires. 

Next, the FBI was induced using two procedures: visuo-motor and visuo-tactile stimulation. Visuo-motor stimulation consisted of synchronizing the movement of the participants and the avatar using motion capture sensors placed on the hands, feet and waist. Once inside the virtual environment, all participants could observe themselves in the first-person perspective and look at themselves in a mirror (in the third-person perspective). The movements were carried out in a structured way, and the procedure lasted one and a half minutes. The visuo-tactile stimulation consisted of synchronizing the participants’ visual and tactile stimulation. When participants were touched with one of the HTC-VIVE controllers on different areas of the body (upper and lower limbs and stomach), they saw (in first and third person) how their avatars were touched in the same areas at the same time by a virtual controller. The visuo-tactile stimulation lasted one and a half minutes and was always performed by a female experimenter. Once the FBI was induced, the three VAS examining the intensity of the FBI, body-related anxiety and the FGW were assessed. 

Finally, to assess the body-related attentional bias, a calibration and recording procedure was conducted using VR HMD-FOVE-Eye Tracking. The participants’ gaze was tracked while they were asked to observe their virtual body in the mirror for 30 s (a similar recording time to that used in other studies) [50,54,55]. During the process, and as a cover story, the participant was told to remain still and avoid abrupt head movements while the virtual avatar position was being recalibrated.

### 2.5. Statistical Analysis

Ogama (Open Gaze Mouse Analyzer) software (Freie Universität, Berlin, Germany) was used to transform ET raw data into suitable quantitative data. The sum of the visual fixation times of each of the subjects was estimated using the complete fixation time and the number of fixations on the AOIs. This was achieved by summing up separate complete fixation times and the number of fixations displayed on W-AOIs vs. on NW-AOIs. Additional data transformation was conducted by calculating the difference between weight-related and non-weight-related AOIs (e.g., complete fixation time (W-AOIs: 1615 ms − NW-AOIs: 1505 ms = 110 ms)). Therefore, a positive outcome would mean that the participant looked at weight-related body parts longer than at non-weight-related body parts, while a negative outcome would mean the opposite. 

Likewise, healthy women were divided into high vs. low BD levels using the median score of the EDI-BD as a cut-off point (_Me_ BD = 8).

One-way analyses of covariance (ANCOVA) were run to determine if there were differences in all measures, including ED measures assessed prior to entering the VR environment, VR-VASs and ET measures between participants with high and low BD and AN patients. Given the age differences between ED and healthy participants, this variable was controlled and introduced as a covariable in the analyses. Analyses of ET measures were conducted for the groups of AOIs (e.g., the difference between W-AOIs and NW-AOIs) and for single W-AOIs (e.g., arms, shoulders and chest, etc.). 

There was homogeneity of the regression slopes as the interaction terms were not statistically significant (*p* > 0.05) for any of the measures. The other assumptions were partially met. There was homogeneity of the variances, as assessed by Levene’s test, in almost all measures except for BMI, EDI-BD, EDI-DT and BIAS-BD. In addition, data were not normally distributed in all the variables assessed by the Kolmogorov–Smirnov test. However, it was decided to conduct the analyses regardless, as ANCOVA is considered a robust test, even in the case of deviation from normality [56]. Finally, a few outliers were detected in some measures, as assessed by inspection of a boxplot. Statistical analyses were conducted with and without the outliers. Since the results did not differ significantly, it was decided to include them in the analyses. 

Finally, Pearson correlations were run to assess the relationship between ED measures, VASs and body-related attentional bias measures in the overall sample. They were also conducted separately among healthy participants with low and high BD and patients with AN. All the analyses were conducted with the statistical software IBM SPSS Statistics v.24 (IBM Corp. Released 2016. Armonk, NY, USA).

## 3. Results

The demographic and clinical information of healthy controls and AN patients are summarized here. The healthy control sample consisted of 43 healthy college women (M_age_ = 21.12, SD = 1.56 and age range: 18–23 years and M_BMI_ = 21.94, SD = 2.53 and BMI range: 17.12 – 27.82), with 25 women with low BD and 18 women with high BD. The clinical sample consisted of 30 AN female patients (M_age_ = 17.73 and SD = 4.60 and M_BMI_ =17.55 and SD =1.07), with 19 adolescents (age range: 13–17 years and BMI range: 16.06–18.94 kg/m^2^) and 11 adults (age range: 18–32 years and BMI range: 13.96–18.98). Of the adolescent AN patients, 18 were diagnosed with restrictive AN (AN-R) and the other one with purgative AN (AN-P). Three adolescent patients presented comorbidities with anxiety disorders: two presented mood-related disorders, and one presented both anxiety and mood-related disorders. Eight patients received pharmacological treatment: two with antidepressants, three with anxiolytics and three with a combination of antidepressants and anxiolytics. 

Six of the adult patients were diagnosed with the AN-P subtype and five with the AN-R subtype. In addition, two adult patients presented comorbidities with borderline personality disorder, one presented borderline personality disorder and post-traumatic stress disorder and one presented a major depressive disorder. Five adult patients were receiving pharmacological treatment: one with antidepressants, one with anxiolytics and antidepressants and three with antidepressants or anxiolytics combined with antipsychotics.

All the descriptive results are summarized in Table 1, including ED measures assessed prior to entering the VR environment, VASs assessed within the VR environment and body-related attentional bias measures. 

### 3.1. Eating Disorder Measures Assessed Prior to Entering the VR Environment

As Table 1 shows, patients with AN reported higher levels of BD, a drive for thinness, body anxiety and body image disturbances (including body distortion and BD assessed with BIAS and EDI questionnaires, respectively) than healthy participants. Patients with AN also had lower BMI and showed lower body appreciation than healthy participants. 

After adjusting for age, the ANCOVA analyses showed group differences on all ED measures. Specifically, the results showed significant group differences in BMI (*F*(2,67) = 29.035, *p* < 0.001, partial η^2^ = 0.464); drive for thinness (*F*(2,69) = 56.775, *p* < 0.001, partial η^2^ = 0.622); BD (*F*(2,69) = 49.101, *p* < 0.001, partial η^2^ = 0.587); body anxiety (*F*(2,67) = 20.600, *p* < 0.001, partial η^2^ = 0.381); body image disturbances (including body distortion: *F*(2,67) = 15.703, *p* < 0.001, partial η^2^ = 0.319 and BD: *F*(2,67) *=* 12,968, *p* < 0.001, partial η^2^ = 0.279, as assessed with the BIAS-BD questionnaire) and body appreciation (*F*(2,67) = 45.426, *p* < 0.001). Post-hoc analyses revealed that there were significant differences (*p* < 0.05) between patients with AN and women with high and low BD in all ED measures. 

### 3.2. VASs of Full-Body Illusion, Body Anxiety and Fear of Gaining Weight

Once in the VR environment, and according to the VASs, AN patients reported lower values of FBI than healthy participants. Additionally, AN patients reported higher levels of body anxiety and FGW, while owning their real-size VB, than healthy participants. Women with high BD also reported higher body anxiety and FGW, as well as lower levels of FBI than women with low BD (see Table 1).

To clarify whether these differences between patients with AN and women with high and low BD were, indeed, significant, one-way ANCOVAs were run. After controlling for age, the results reported significant group differences in FBI levels (*F*(2,68) = 6.252, *p* = 0.003, partial η^2^ = 0.157); FGW (*F*(2,67) = 16,670, *p* < 0.001, partial η^2^= 0.332) and marginally significant differences in body anxiety (*F*(2,67) = 3.077, *p* = 0.053, partial η^2^ = 0.084). Effect sizes according to Cohen (1988) were medium in body anxiety, high in FBI and very high in FGW. Post-hoc analyses revealed significant differences (*p* < 0.05) between patients with AN and women with low BD in FBI and FGW VASs and marginally significant differences (*p* = 0.057) in VAS-A (see Table 2). In addition, there were also significant differences between women with high BD and low BD (see Table 2). 

### 3.3. Body-Related Attentional Bias Measures

The descriptive results of both ET measures revealed that AN patients spent more time looking at and showed a higher number of fixations on weight-related AOIs than on non-weight-related AOIs, as indicated by a positive outcome in both ET measures. In contrast, women with high BD spent a similar time and number of fixations looking at weight- and non-weight-related AOIs. Finally, women with low BD showed a tendency to spend more time looking at NW-AOIs and a similar number of fixations on weight- and non-weight-related AOIs (see Table 1). 

The one-way ANCOVA results showed statistically significant group differences in the complete fixation time (*F*(2,63) = 13.114, *p < 0.001*, partial η^2^ = 0.294) and the number of fixations (*F*(2,63) = 17.107, *p* < 0.001, partial η^2^ = 0.352) after controlling for age. Post-hoc analyses revealed that these differences were statically significant (*p* < 0.05) between patients with AN and women with high and low BD in both attentional bias measures (see Table 2). Finally, there were no significant differences (*p* > 0.05) between women with high and low BD.

**Figure 2:**
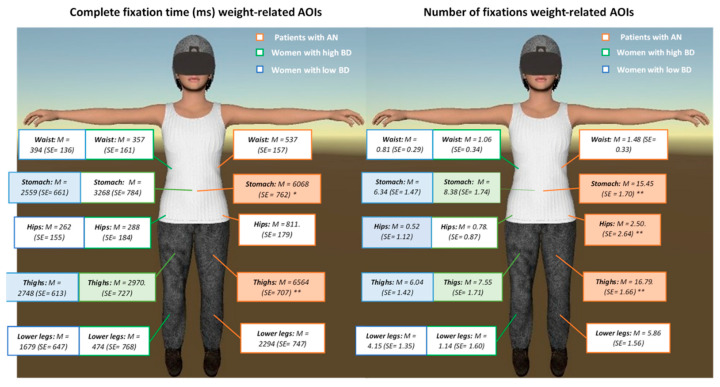
Differences between patients with Anorexia Nervosa (AN) and women with high and low body dissatisfaction (BD) (means and standard errors) in the complete fixation time (in milliseconds; ms) and the number of fixations on individual weight-related areas of interest (AOIs). Post-hoc analyses: ** *p* < 0.01 and * *p* < 0.05.

One-way ANCOVAs were also run to assess the group differences at single W-related AOIs. After controlling for age, the results showed statistically significant group differences (*p* < 0.05) for the thighs (complete fixation time: *F*(2,63) = 7707, *p* = 0.001, partial η2 = 0.197 and number of fixations: *F*(2,63) = 10.573, *p* < 0.001, partial η2 = 0.251); hips (number of fixations: *F*(2,63) = 4663, *p* < 0.013, partial η2 = 0.129) and stomach (complete fixation time: *F*(2,63) = 5239, *p* = 0.008, partial η2 = 0.143 and number of fixations: *F*(2,63) = 7078, *p* = 0.002, partial η2 = 0.183). Post-hoc analyses revealed that patients with AN spent significantly more time looking and showed higher numbers of fixations on the stomach, hips and, particularly, the thighs in contrast to women with low and high BD (Figure 2). No significant differences were found between women with high and low BD for any of the single AOIs.

### 3.4. Pearson Correlations

According to multiple Pearson correlations, the results showed statistically significant moderate or large positive correlations between VAS-A and VAS-FGQ and all ED measures (*p* < 0.05), which were significantly larger for VAS-FGW (see Table 3). There were also moderate or large positive correlations between VAS-A/FGW and the BMI and BAS scale. Furthermore, VAS-FBI negatively correlated (*p* < 0.05) with the VAS-FGW, EDI-DT, EDI-BD, BAS and BIAS-BD measures, with moderate correlations (Cohen, 1988). There was a moderate positive correlation between the VAS-FBI and BAS. Finally, there were significant moderate positive correlations between the body-related attentional bias measures and the EDI-DT, EDI-BD and PASTAS (only for the number of fixations (NF)) scales, while there were moderate negative correlations between the body-related attentional bias measures and the BMI and BAS.

Multiple Pearson correlations were conducted separately for women with low and high BD and for patients with AN. The results among women with low BD showed that, overall, the previously reported positive and negative correlations between the VASs/attentional bias measures and other ED measures were not significant, with the exception of the VAS-FGW and PASTAS/BAS (see Table 4). Similar results were found among women with high BD, in which there were statistically significant positive/negative correlations between the VAS-FGW and BMI, EDI-DT, PASTAS and BAS (See Table 5). Finally, among patients with AN, there were statistically significant and similar positive and negative correlations to those previously reported for the entire clinical and nonclinical samples (see Table 6).

Surprisingly, some of the strongest significant negative correlations were found in FBI levels and body image disturbance measures only among patients with AN. To explain these preliminary results, it was hypothesized that the individuals with higher body image disturbance (i.e., body distortion/body dissatisfaction) levels were those who experienced lower FBI levels once they owned their real-size virtual body. Thus, it was decided to conduct predictive post-hoc linear regression analyses. The results showed that having higher body distortion levels statistically significantly predicted experiencing lower FBI levels (*F*(1,29) = 6.160, *p* = 0.019), accounting for 18% of the explained variability in FBI levels only among patients with AN. This relationship between body distortion and FBI levels was not significant among women with high and low BD (*p* > 0.05). Similarly, experiencing higher BD levels significantly predicted experiencing lower FBI levels (*F*(1, 29) = 6.561, *p* = 0.016), which accounted for 19% of the explained variability in FBI levels only among patients with AN. Again, the relationship between body distortion and FBI levels was not significant among women with high and low BD (*p* > 0.05).

## 4. Discussion

The current study aimed to provide initial evidence of the usefulness of VR body exposure to elicit FGW, body anxiety and body-related attentional bias in patients with AN. For this purpose, we assessed whether there were significant differences in the levels of FBI, body anxiety, FGW and body-related attentional bias when AN patients and healthy women with high and low BD owned a virtual body with their real silhouette and body mass index (BMI). As expected, patients with AN showed higher levels of FGW, body anxiety and body-related attentional bias than healthy controls when they owned their real-size VB. Unexpectedly, patients with AN reported significantly lower levels of FBI than women with low BD. Furthermore, no significant differences were found between healthy women with high and low BD on FBI, body anxiety or body-related attentional bias. The only significant group differences were observed in the levels of FGW reported.

Furthermore, the relationship between the ED measures assessed prior to entering the VR and the responses produced during VR exposure was also assessed. As expected, there were positive relationships between some measures assessed by VR body exposure (e.g., body anxiety and FGW) software and other ED measures assessed previously. This relation was particularly strong among patients with AN. Hence, the participants and patients with higher ED symptomatology were also those who experienced higher levels of FGW, body anxiety and attentional bias during exposure. Additionally, significant negative relationships were found among FBI and body image disturbance measures, including body distortion and BD, only among patients with AN.

According to the measures assessed during the VR body exposure, patients with AN reported significantly higher FGW and body anxiety levels than women with high and low BD, with high effect sizes. Specifically, the FGW was the variable that best distinguished between AN patients and women with low BD and between women with high and low BD. Relatedly, the FGW during VR body exposure was the measure that had the largest significant correlations with the ED measures assessed before exposure, such as BD, drive for thinness, body anxiety, BMI and body appreciation. Similar tendencies were observed not only among patients with AN but, also, among women with high BD. These results support the increasing evidence in favor of the critical role that the FGW might display not only in AN [6,8,9,10] but, also, in healthy women with body concerns. Indeed, several studies have reported high prevalence of FGW among adult women [2,57] and young women between 16 to 25 years old [58].

Unexpectedly, patients with AN reported significantly lower FBI levels than healthy participants with low BD. Our results seem to contradict previous research that found that ED patients tend to experience a stronger illusion than healthy individuals [34]. However, those studies induced the feeling of ownership in one hand only, following the rubber hand illusion paradigm [33]. In the current study, the illusion of ownership was induced over a whole virtual body, which suggests that other body-related factors (e.g., body image disturbances) could influence these results as well. For instance, several patients with AN described their virtual body as being slimmer than their real body, while healthy women with high and low BD did not report such a phenomenon. Accordingly, there was a significant negative relationship between FBI and body image disturbances only among patients with AN, while this relationship was not significant among women with high and low BD. After further analyses, it was found that AN patients with higher body distortion and body dissatisfaction levels experienced lower FBI levels once they owned their real-size virtual body. These results provide preliminary evidence about the influence that body image disturbances have on FBI over the whole body among patients with AN. Accordingly, two interesting questions should be considered. Firstly, would these low FBI levels increase during a body exposure treatment, for instance, over several sessions? Secondly, if this were the case, would a continuous increase in FBI levels produce a change in body image disturbances in the participants or the other way around? Unfortunately, it is not possible to answer either question, since there is a lack of studies in which FBI levels were assessed over several sessions with AN patients. In fact, to the best of our knowledge, only one study has assessed FBI over a short period among healthy participants, finding that, after a synchronous visuo-motor embodiment procedure, FBI levels remained high after 30 and 55 s of synchronous or no movement [59].

Regarding body-related attentional bias measures, our results agree with previous studies that suggest that women with EDs tend to pay more attention to self-defined unattractive body areas, while healthy participants tend to show more general scanning behavior, covering the whole body [17,54,60,61]. While most of the studies assessing body-related attention have been conducted with adults, little information is available about body-related attention in youths. Therefore, our results also support studies that assessed body-related attentional bias among adolescents with EDs and showed that adolescents with AN and Bulimia Nervosa spent more time on self-reported unattractive body parts [18,62]. For instance, Bauer et al. [18] found that dysfunctional body-related attention was more marked among adolescents with AN-R than among healthy individuals and other ED patients (e.g., patients with AN-binge eating/purging subtype or Bulimia Nervosa). Since almost all our patients were diagnosed with an AN-R subtype, this study reinforced the evidence of dysfunctional body-related attention among adolescents with AN-R. When single W-related AOIs were considered, we observed that all individuals paid more attention to the thighs, stomach and lower legs. The W-AOIs that best distinguished between patients with AN and women with high and low BD were the thighs, the stomach, and the hips (only in number of fixations). This suggests that these areas might be particularly salient for patients with AN.

Nevertheless, some key methodological differences between the current study and other studies that assess body-related attentional bias should be mentioned. As described before, some previous studies used a similar free-viewing, single-body paradigm but measured the attentional bias towards self-reported attractive vs. unattractive body areas. This is a successful, well-established methodology to define the areas of interest [16,17]. Although self-reported unattractive body areas among individuals with EDs could indeed be areas related with weight, the evidence for attentional bias towards specific weight-related body areas could not be clearly established, since there might be weight-related body areas in both the self-reported attractive and unattractive body parts. In this study and in previous studies conducted by our group, a different methodology was used to define the areas of interest, in which an individual’s gazing behavior was analyzed using the same definition of areas of interest for all participants e.g., weight- vs. non-weight-related body areas [50,51]. These areas of interest were defined based on well-established questionnaires that assess the same body image construct, such as the weight-related scale of the PASTAS.

Finally, no differences in body-related attentional bias were found between women with high and low BD. Women with high BD tended to pay more attention to W-related AOIs, while women with low BD showed the opposite visual tendency toward NW-related AOIs. None of these tendencies showed a clear attentional bias to weight- or non-weight-related AOIs. However, overall, both groups showed more general scanning behavior, covering the whole body. These results are in-line with a previous study conducted in our group [50] in which women with high and low BD showed similar attentional patterns toward their own virtual bodies. This previous study was conducted with a previous version of the virtual simulation. Although the results obtained were very similar, some technical improvements that were made to the current version of the simulation should be considered. For instance, a more precise method could be used to create the avatar (by taking a frontal and lateral photo and incorporating the BMI information of each participant), and the full-body tracking system could be improved to conduct a visual-motor stimulation.

Some limitations should be considered in the current study. For instance, most of the patients with AN were adolescents (19 out of 30), while the healthy women were college students between 18 and 22 years old. Although the potential effect of age between groups was controlled in this study by ANCOVAs, these statistical analyses could not control the effect that age might play within each group and, particularly, in the more heterogeneous AN sample. Future studies should try to overcome this important bias and replicate the current study with age-balanced samples.

Regarding the assessment, no screening questionnaires or structured clinical interviews were used properly to assess the presence of EDs or other mental disorders among the healthy participants. Furthermore, to diagnosticate the clinical sample, a MINI diagnostic interview was used in two of the three ED units, while, at the last ED unit, senior trained clinical psychologists and psychiatrists conducted nonstructured clinical interviews following thoroughly the DSM-5 criteria.

FBI and FGW were assessed using a VAS; even though VASs are usually considered as a valid measure to assess this sort of construct (for instance, FGW among patients with AN [63]), they should be complemented with evidence-based questionnaires. For instance, the embodiment questionnaire [64] might offer a more accurate assessment of the FBI based on the location of the body, the ownership illusion, the motor agency and the general appearance. One of the reasons for using the VAS was to assess the FBI while patients and participants were owning their real-size virtual bodies. It was important to assess the FBI levels directly in the VR environment and not later, since this bodily illusion might have been reduced once the participants left the VR (after the ET assessment task), and the results might have been affected as a consequence. However, since several questionnaires were already used in the current research, it was decided not to extend the assessment sessions (which lasted approximately 1h) any further and to refrain from applying larger tests. Future studies might try to implement evidence-based questionnaires that assess these two measures. Finally, in the current study, the average levels of BD among women labeled as “high BD” were very similar to in a previous study conducted by our group [50], corresponding to medium-to-high BD levels among nonclinical Spanish women [44]. Thus, it would have been necessary to recruit participants with higher levels of BD to provide a more exhaustive distinction of the BD levels among women with high and low BD. Relatedly, the suitability of using the median as an “artificial categorization” of a continuous variable—in this study, BD—could have some limitations. These could affect the statistical power and accuracy of the estimated relations and reduce the observed relations among the variables [65].

Some limitations in the VR software should also be considered. Although several improvements were implemented (e.g., the avatar sharing the same silhouette and BMI as the participant), the general appearance of the virtual body (clothes and skin color, etc.) was not exactly that of the individuals. The latest VR studies allow the simulation of an exact 3D biometric avatar with all the individual’s features [66] using 3D body scans. This sort of technology might notably enhance the realism of VR embodiment-based techniques and, consequently, improve VR studies on body-related issues and EDs. The current study used two separate HMDs: one for conducting the visuo-tactile and visuo-motor FBI procedures (HTC-VIVE) and the other to record the body-related attentional bias measures (FOVE-VR). Having two separate devices might have reduced the FBI levels when the participants had to change from one HMD to the other. This limitation could have been overcome by using the new generation of VR HMD with ET devices all-in-one (e.g., the HTC VIVE-Pro Eye, which has a complete full-body tracking system and can be used to elicit FBI over the virtual body and measure gaze patterns).

The implications of VR embodiment-based techniques might be critical to improving evidence-based therapies in AN, such as body exposure-based techniques. For instance, instead of exposing a patient with AN to food or to her current body image, as has been done so far, what would be the result of exposing her to her core fear, i.e., weight gain? Patients with AN associate weight gain with negative consequences, and food avoidance prevents them from learning that maintaining a normal weight does not lead to such catastrophic consequences [67]. Consequently, focusing on the primary conditioned stimulus (weight gain) rather than on the secondary ones, such as eating, may be an effective strategy. As has been reported, VR embodiment-based techniques are a successful way to change body image disturbances in patients with AN [39,40,41]. We propose going one step further. First, we could allow the patient with AN to experience the illusion of ownership of a virtual body that reproduces their real-size silhouette, with their specific physical particularities and their exact BMI. Then, we could apply progressive increases in weight (or an increase in the BMI) of the virtual avatar, until it reaches a healthy BMI.

Furthermore, the combination of VR and ET technologies to assess body-related attentional bias might lead to new possibilities in coming years. For instance, it might improve some of the current limitations of research conducted with fixed ET, such as the lack of external validity [68]. In other words, combining ET and VR might enable the reproduction of more ecological environments, such as bathrooms and dressing rooms, to assess patient’s gaze patterns while they own their real-size virtual avatars in each session or before and after the therapy.

To sum up, our results show that, while owning their real-size virtual body, patients with AN reported significantly higher FGW and body anxiety levels than women with low BD, with high effect sizes. Specifically, FGW was the variable that best distinguished between AN patients and healthy participants and between healthy participants with high and low BD. Unexpectedly, patients with AN reported significantly lower FBI levels than healthy participants with low BD. After post-hoc analyses, it was found that body image disturbances influence FBI levels among patients with AN. Furthermore, our results suggest that the patients with AN showed a body-related attentional bias. Specifically, they spent more time and looked more frequently at the weight-related body areas (e.g., thighs, stomach and hips) than healthy women. Finally, these results provide evidence about the usefulness of VR-based body exposure to elicit FGW and other body-related disturbances in AN patients. Thus, it may be a suitable intervention for reducing these emotional responses and for easing weight recovery.

## Figures and Tables

**Table 1:** Adjusted for age and unadjusted descriptive results. Mean (M), standard deviation (SD) and standard error (SE).

	Women with Low BD (*n*= 25)		Women with High BD (*n*= 18)		AN Patients (*n* = 30)	
	Unadjusted	Adjusted	Unadjusted	Adjusted	Unadjusted	Adjusted
	M (SD)	M (SE)	M (SD)	M (SE)	M (SD)	M (SE)
BMI	21.65 (2.39)	21.60 (.42)	22.38 (2.75)	22.32 (.53)	17.55 (1.07)	17.63 (0.40)
EDI-3-DT	1.88 (1.76)	2.41 (1.07)	4.89 (4.39)	5.57 (1.27)	19.23 (7.43)	18.38 (1.03)
EDI-3-BD	4.04 (1.44)	4.53 (1.32)	14.61 (6.30)	15.26 (1.56)	24.03 (10.03)	23.23 (1.27)
PASTAS	5.04 (3.77)	5.80 (1.09)	8.56 (4.77)	9.70 (1.38)	17.20 (7.18)	15.95 (1.05)
BIAS-Body distortion	9.60 (13.06)	9.93 (3.59)	18.13 (13.76)	18.61 (4.51)	39.17 (21.45)	38.63 (3.43)
BIAS-Body dissatisfaction	7.40 (9.90)	7.53 (4.02)	14.37 (19.56)	14.56 (5.07)	36.83 (24.62)	36.62 (3.86)
BAS	52.32 (5.29)	51.82 (1.57)	42.06 (7.54)	41.32 (1.98)	29.43 (9.30)	30.24 (1.51)
VAS-FBI	65.72 (23.25)	67.94 (5.05)	54.94 (19.20)	58.24 (6.38)	46.18 (29.50)	42.15 (4.86)
VAS-A	15.20 (19.12)	17.44 (5.15)	26.25 (22.97)	29.60 (6.51)	40.00 (31.15)	35.34 (4.95)
VAS-FGW	22.88 (28.80)	25.12 (5.84)	51.88 (26.39)	55.21 (7.38)	79.82 (27.70)	72.86 (5.62)
Complete fixation time (in ms) *	−2858 (10,589)	−4492 (1732)	965 (7578)	−1079 (2055)	6899 (7457)	10,198 (2014)
Number of fixations *	2.20 (15.80)	−0.45 (2.96)	5.20 (15.49)	1.90 (3.51)	22.00 (12.99)	27.25 (3.41)

Note: body mass index (BMI); Eating Disorder Inventory (EDI-3) drive for thinness (DT) and body dissatisfaction (BD) scales; Physical Appearance State and Trait Anxiety Scale (PASTAS); Body Image Assessment Test (BIAS); Body Appreciation Scale (BAS) and visual analog scales (VAS) of full body illusion (FBI), body anxiety (A) and fear of gaining weight (FGW). AN = Anorexia Nervosa. Complete fixation time in milliseconds (ms). * For eye-tracking (ET) measures, the clinical sample was comprised of 19 adolescents and 5 adults with AN.

**Table 2:** Post-hoc analyses (pairwise comparison) between AN patients and women with high and low BD.

Measures	AN Patients vs. Women with Low BD			AN Patients vs. Women with High BD			Women with High vs. Low BD		
	MD	*p*	95% CI	MD	*p*	95% CI	MD	*p*	95% CI
VAS-FBI	−25.79	0.002	(−43.72, −7.86)	−16.10	0.179	(−36.73, 4.53)	−9.69	0.672	(−29.08, 9.70)
VAS-A	17,89	0.057	(−0.39, 36.18)	5,74	0.896	(−15.30, 26.78)	12,15	0.408	(−7.62, 31.93)
VAS-FGW	47.74	<0.001	(68.48, 27.00)	17.64	0.221	(−6.21, 41.51)	30.09	0.005	(7.66, 52.52)
Complete fixation time (in ms) *	14,722	<0.001	(7626, 21,818)	11,317	0.002	(3590, 19,045)	3404	0.568	(−2904, 9712)
Number of fixations *	27.70	<0.001	(15.66, 39.74)	25.35	<0.001	(12.24, 38.45)	2.35	0.787	(−8.34, 13.05)

Note: Mean differences (MD), *p*-values and 95% confidence intervals (CI) stated for each group comparison. ms = milliseconds. * For ET measures, the clinical sample was comprised of 19 adolescents and 5 adults with AN.

**Table 3:** Correlations between eating disorder (ED) measures and virtual reality (VR) body exposure responses in the entire sample.

	VAS-FBI	VAS-A	VAS-FGW	CFT	NF
VAS-FBI					
VAS-A	−0.16				
VAS-FGW	−0.24 *	0.70 **			
CFT	−0.21	0.16	0.35 **		
NF	−0.28	1.18	0.42 **	0.91 **	
BMI	0.23	−0.22	−0.38 **	−0.38 **	−0.43 **
EDI-DT	−0.37 **	0.55 **	0.72 **	0.32 **	0.41 **
EDI-BD	−0.30 **	0.53 **	0.73 **	0.35 **	0.40 **
PASTAS	−0.22	0.60 **	0.80 **	0.22	0.32 **
BIAS-Body distortion	−0.43 **	0.38 **	0.56 **	0.16	0.21
BIAS-Body dissatisfaction	−0.38 **	0.28 *	0.50 **	0.19	0.23
BAS	0.34 **	−0.58 **	−0.77 **	−0.32 **	−0.40 **

* = *p*-values < 0.005 or ** = *p*-values < 0.001. NOTE: CFT = complete fixation time. NF= number of fixations.

**Table 4:** Correlations between ED measures and VR body exposure responses in healthy participants with low BD.

	VAS-FBI	VAS-A	VAS-FGW	CFT	NF
VAS-FBI					
VAS-A	−0.028				
VAS-FGW	−0.209	0.778 **			
CFT	−0.290	−0.097	0.205		
NF	−0.255	0.064	0.402 *	0.938 **	1.00
BMI	−0.020	0.071	−0.013	−0.277	−0.243
EDI-DT	−0.268	0.279	0.325	0.235	0.246
EDI-BD	−0.045	0.153	0.172	0.316	0.336
PASTAS	−0.057	0.728 **	0.686 **	−0.045	0.015
BIAS-Body distortion	−0.147	0.142	−0.085	−0.155	−0.199
BIAS- Body dissatisfaction	0.239	0.195	−0.041	−0.149	−0.099
BAS	−0.201	−0.503 *	−0.593 **	−0.214	−0.322

* = *p*-values < 0.05 or ** = *p*-values < 0.01. NOTE: CFT = complete fixation time. NF = number of fixations.

**Table 5:** Correlations between ED measures and VR body exposure responses in healthy participants with high BD.

	VAS-FBI	VAS-A	VAS-FGW	CFT	NF
VAS-FBI					
VAS-A	0.173				
VAS-FGW	0.282	0.710 **			
CFT	0.300	0.040	0.017		
NF	0.433	0.183	0.006	0.876 **	
BMI	0.170	−0.045	0.016	−0.096	0.009
EDI-DT	0.086	0.271	0.519 *	−0.241	−0.281
EDI-BD	0.035	0.452	0.546 *	0.071	0.132
PASTAS	0.077	0.719 **	0.753 **	0.030	0.057
BIAS-Body distortion	−0.103	0.171	0.377	0.041	−0.143
BIAS-Body dissatisfaction	−0.108	−0.246	0.048	0.040	−0.093
BAS	−0.162	−0.350	−0.674 **	0.053	0.099

* = *p*-values < 0.05 or ** = *p*-values < 0.01. NOTE: CFT = complete fixation time. NF = number of fixations.

**Table 6:** Correlations between ED measures and VR body exposure responses in patients with AN.

	VAS-FBI	VAS-A	VAS-FGW	CFT	NF
VAS-FBI					
VAS-A	−0.093				
VAS-FGW	−0.031	0.563 **			
CFT	−0.057	−0.187	−0.191		
NF	−0.064	−0.317	−0.340	0.862 **	
BMI	0.042	0.116	0.059	0.141	0.149
EDI-DT	−0.290	0.562 **	0.620 **	−0.326	−0.263
EDI-BD	−0.107	0.442^*^	0.594 **	−0.211	−0.206
PASTAS	0.014	0.399 *	0.675 **	−0.527 *^*^	−0.472 *
BIAS-Body distortion	−0.425 *	0.216	0.458 *	−0.280	−0.220
BIAS-Body dissatisfaction	−0.436 *	0.163	0.396 *	−0.164	−0.172
BAS	0.351	−0.550 **	−0.564**	0.377	0.321

* = *p*-values < 0.05 or ** = *p*-values < 0.01. NOTE: CFT = complete fixation time. NF = number of fixations.
